# Genome-wide development of insertion-deletion (InDel) markers for Cannabis and its uses in genetic structure analysis of Chinese germplasm and sex-linked marker identification

**DOI:** 10.1186/s12864-021-07883-w

**Published:** 2021-08-05

**Authors:** Gen Pan, Zheng Li, Siqi Huang, Jie Tao, Yaliang Shi, Anguo Chen, Jianjun Li, Huijuan Tang, Li Chang, Yong Deng, Defang Li, Lining Zhao

**Affiliations:** 1 Institute of Bast Fiber Crops, Chinese Academy of Agricultural Sciences, Changsha, 410205 China; 2 Key Laboratory of the Biology and Process of Bast Fiber Crops, Ministry of Agriculture, Changsha, China

**Keywords:** Cannabis, Insertion–deletion (InDel), Population structure, Sex identification

## Abstract

**Background:**

*Cannabis sativa* L., a dioecious plant derived from China, demonstrates important medicinal properties and economic value worldwide. Cannabis properties have been usually harnessed depending on the sex of the plant. To analyse the genetic structure of Chinese Cannabis and identify sex-linked makers, genome-wide insertion-deletion (InDel) markers were designed and used.

**Results:**

In this study, a genome-wide analysis of insertion-deletion (InDel) polymorphisms was performed based on the recent genome sequences. In total, 47,558 InDels were detected between the two varieties, and the length of InDels ranged from 4 bp to 87 bp. The most common InDels were tetranucleotides, followed by pentanucleotides. Chromosome 5 exhibited the highest number of InDels among the Cannabis chromosomes, while chromosome 10 exhibited the lowest number. Additionally, 31,802 non-redundant InDel markers were designed, and 84 primers evenly distributed in the Cannabis genome were chosen for polymorphism analysis. A total of 38 primers exhibited polymorphisms among three accessions, and of the polymorphism primers, 14 biallelic primers were further used to analyse the genetic structure. A total of 39 fragments were detected, and the PIC value ranged from 0.1209 to 0.6351. According to the InDel markers and the flowering time, the 115 Chinese germplasms were divided into two subgroups, mainly composed of cultivars obtained from the northernmost and southernmost regions, respectively. Additional two markers, “Cs-I1–10” and “Cs-I1–15”, were found to amplify two bands (398 bp and 251 bp; 293 bp and 141 bp) in the male plants, while 389-bp or 293-bp bands were amplified in female plants. Using the two markers, the feminized and dioecious varieties could also be distinguished.

**Conclusion:**

Based on the findings obtained herein, we believe that this study will facilitate the genetic improvement and germplasm conservation of Cannabis in China, and the sex-linked InDel markers will provide accurate sex identification strategies for Cannabis breeding and production.

**Supplementary Information:**

The online version contains supplementary material available at 10.1186/s12864-021-07883-w.

## Introduction

*Cannabis sativa* L., a member of the family Cannabinaceae, is a diploid (2n = 20) monocotyledon and one of the oldest cultivated plants. Although it originated in Central Asia, its cultivation was soon commenced worldwide for applications in folk medicine, textile fibre, oil, and recreational use [1]. Cannabis is a botanical genus of flowering plants divided into two distinct species, namely Hemp and marijuana, based on its tetrahydrocannabinol (THC) content [2]. Although Cannabis cultivation is being restricted in many countries due to its widespread usage as a recreational drug, there has been a resurgence of interest for its agronomic potential and especially its medical value; its outer and inner stem tissues can be used to prepare bioplastics and concrete-like material in construction sectors owing to the rich source of both cellulosic and woody fibres, and its metabolites exert potent bioactivities on human health especially for the treatment of pediatric seizure disorders.

Cannabis is a dioecious species, which includes both male and female flowers separated on different plants. The sex of the plants commonly affects economically relevant traits like fibre quality and cannabinoid (CBD) content. In general, male plants have a better fibre quality, while CBD content in female plants is higher than that in male plants. Therefore, an ideal ratio of male-to-female individuals must be maintained with different production purposes to improve economic efficiency. However, it is difficult to identify the sex of plant via the mere examination of morphological traits before flowering, and DNA molecular marker technology has been considered as an accurate and reliable method for the sex identification of dioecious plants, as it is unaffected by plant growth stages [3].

Conventional breeding is considered the primary method for developing new varieties in Cannabis breeding programs. However, this process is extremely challenging and often spans several years [4]. Previous studies have indicated that advancements in molecular technologies offer several molecular breeding strategies, such as the use of molecular markers to overcome the limitations of conventional breeding [4, 5]. A shift from isozyme and random amplified polymorphic DNA (RAPD) to amplified fragment length polymorphism (AFLP), simple sequence repeat (SSR), and single nucleotide polymorphism (SNP) has occurred, and these markers have been used for genetic analysis and sex identification in Cannabis [6–15]. Although different types of Cannabis molecular markers have been identified and utilized, research on Cannabis is lagging compared to other crops like rice, wheat, and maize. As a result, the density of molecular markers in Cannabis is relatively low, which is insufficient for genetic study in Cannabis, including genetic map construction, gene/QTL mapping, and genetic analysis.

Insertion–deletions (InDels) are recognised as major sources of genetic structural variations found widely distributed across the plant genomes. InDels like SSRs are also a type of length polymorphisms originating from a single mutation event, which is generally bi-allelic and single-locus in nature. Meanwhile, InDels exhibit many desirable inherent genetic characteristics of both SNP and SSR markers, such as co-dominance, abundance, and random distribution across the genome [16]. Generally, unlike SNP, InDel markers have been considered breeder-friendly markers, with limited infrastructure requirements, and its products can be detected in regular genetics and breeding laboratories using polyacrylamide gel electrophoresis (PAGE) or simple gel-based size separation procedures. Furthermore, InDels markers are commonly amplified without stutter bands, which renders them more valuable. In a few previous studies, InDels were also found to be more polymorphic than microsatellite markers [17, 18]. As a valuable complement for both SNPs and SSRs markers and owing to their significance in crop genomic studies, InDel markers have been widely identified in rice [19], barley [20], oil rapes [18, 21], maize [22], and other plants [23–26], and to our knowledge, no research on genome-wide development of InDels in Cannabis has been reported so far. This knowledge gap limits the comprehensive molecular analysis of Cannabis.

China has been considered one of the putative centres of origin for Cannabis, and a region where Cannabis has been cultivated for more than 2000 years for obtaining fibre, oil, and for other purposes [27]. However, the fibre yield, fibre quality, and CBD content are vital factors limiting the development of the Cannabis industry in China, rendering significance to the genetic improvement of the Cannabis crop cultivated in China. Previous studies have shown that the genetic structure analysis of the germplasm can facilitate genetic improvement in other crops [28, 29]. Until now, the genetic diversity and population structure of Cannabis were analysed using SSR and ISSR markers [9, 10, 30, 31]. However, in Cannabis, most SSR and ISSR markers usually display multiple loci [9, 10, 30, 31], thereby posing challenges in the application of molecular analysis such as the comparison of genes/QTLs detected using different genetic populations in Cannabis. Alternatively, the single-locus nature of InDels may help overcome this drawback of multi-locus SSRs and ISSRs. Though the draft genome sequences data were published in 2011 [32], data quality has not met the criteria for genome-wide development of InDel markers and the location of such valuable markers in the Cannabis chromosome has not been elucidated. Recently, a high-quality chromosome-scale reference genome of a drug-type strain “Purple Kush” and the hemp variety “Finola” were obtained, which enabled the genome-wide capture of InDels in the Cannabis genome [33]. With the objectives to increase the density of molecular markers of Cannabis genome and to establish a significance for SSR markers in Cannabis genomic studies, the present study focused on the genome-wide development of InDels and the application of these markers in genetic structure analysis of Chinese germplasm and identification of sex-linked marker in Cannabis. Our study results will help establish a valuable tool for the molecular analysis of Cannabis in the future, and the information on the genetic structure of the Cannabis germplasm and sex-linked marker will aid the genetic improvement and molecular breeding of Cannabis.

## Result

### Distribution of InDel markers

**Fig. 1:**
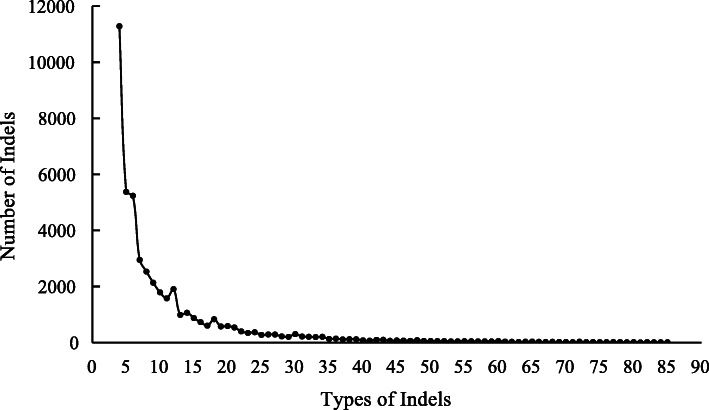
Frequency analysis of InDels type based on the sequence of two accessions (FN and PK)

**Fig. 2:**
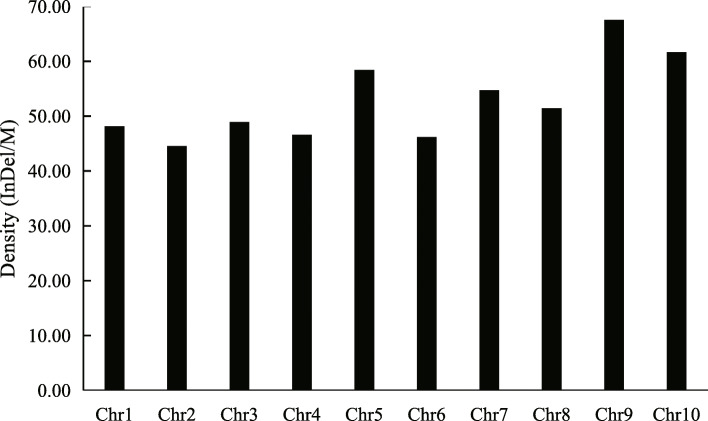
The number of Insert/Deletion (InDel) repeats on 10 chromosomes of Cannabis genome

**Fig. 3:**
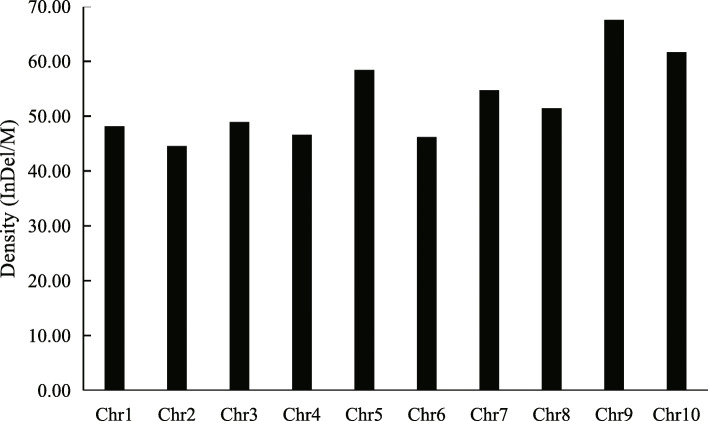
Distribution of Insert/Deletion (InDel) density in Cannabis chromosome

Data on whole genomes for “Purple Kush” and “Finola” were downloaded from ftp://ftpmips.helmholtz-muenchen.de/plants/barley/public_data/. On a genome-wide basis, 47,558 InDels were identified between PK and FN in the genomic DNA sequence database (Table S1). InDel sites varied from 4 bp to 87 bp, and the number of the InDel sites decreased markedly with an increase in the InDel length. Four InDel sites were found to be the most common InDel sites (11286), accounting for 23.7% of the total InDels (Fig. 1). Meanwhile, the distribution of the InDels on each chromosome of the FN genome was different. As shown in Fig. 2, the number of InDels on each chromosome ranged from 2177 to 5081. Chromosome 5 exhibited the highest number of InDels among the Cannabis chromosomes, while chromosome 10 exhibited the lowest. Additionally, the densities of InDels on each chromosome were different, and chromosome 9 exhibited the highest density of InDels (67.5 InDels/Mb) while chromosome 2 exhibited the lowest (44.5 InDels/Mb) (Fig. 2, Fig. 3).

### Development of InDel markers for whole Cannabis genome and polymorphism analysis

In total, 47,558 InDel markers between FN and PK were successfully developed, with a density of 47.1/Mb in the FN genome. Of these InDel markers, 31,802 InDel markers were non-redundant based on the specificity, and its density in the FN genome was found to be 31.51/Mb (Table S1). The lengths of all primers ranged between 18 bp and 24 bp, and the product sizes ranged from 80 bp to 400 bp. Eighty-four primer pairs distributed along the chromosomes with intervals of about 10 Mb were selected to evaluate the quality of InDel markers across three Cannabis varieties (Fig. S1). The results showed that 80 primers were amplified successfully, and 38 primers exhibited polymorphisms among three varieties (“Yunma 6”, “Neimengudali”, “Qingdama 1”). Of all the polymorphism primers, 14 primers which exhibited two alleles among the above-mentioned three varieties were used for further study.

### Genetic diversity analysis and population structure

**Fig. 4:**
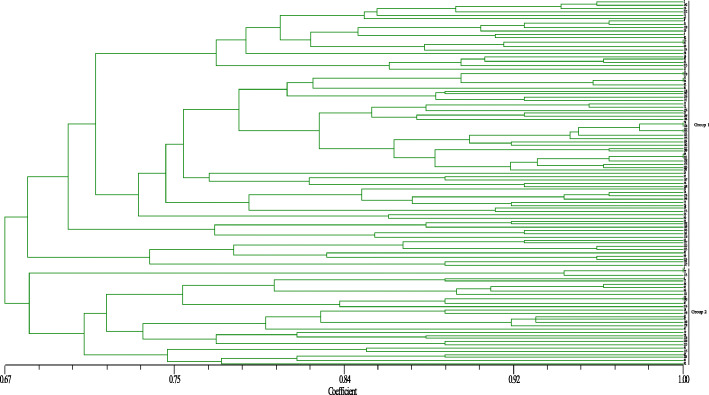
Dendrogram generated by UPGMA cluster analysis of 115 genotypes of Cannabis based on 14 polymorphic genic InDel markers

The 14 InDel primers were used to analyse the genetic relationships of 115 accessions, and a total of 39 polymorphic bands were amplified. The PIC ranged from 0.1209 to 0.6351, with an average of 0.4109, and the gene diversity varied from 0.1243 to 0.6865, with an average of 0.4664. The average MAF was 0.6484 and ranged from 0.4478 to 0.9348 (Table 2). Thereafter, cluster analysis was conducted based on the unweighted pair-group method with arithmetic means (UPGMA) using the NTSYS-pc2.11 software. As showed in Fig. 4, at a genetic distance of 0.74, the 115 accessions were divided into two groups. Group I included 84 accessions, mainly consisting of the varieties cultivated in the northern regions of China (up to 90%). Group II included 31 accessions, and most of them were from the southern regions of China (90.3%).

**Fig. 5:**
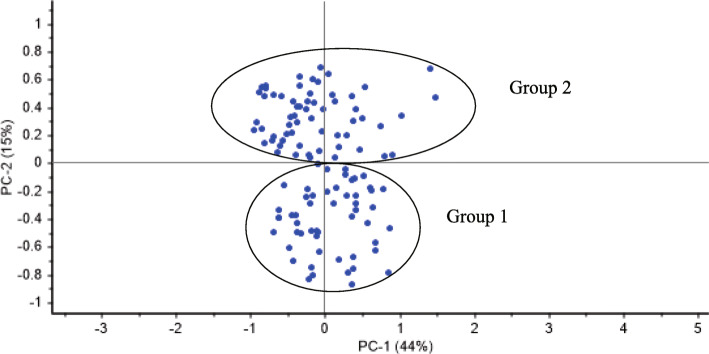
Principles coordinate analysis for InDel markers using the genetic similarity matrix for 115 Chinese Cannabis cultivars

In PCoA, the two main axes explained approximately 59% of the total variation, at 44 and 15%, respectively. The 115 Cannabis varieties could also be classified into two groups using the genetic similarity matrix, which was similar to cluster analysis results (Fig. 5).

**Fig. 6:**
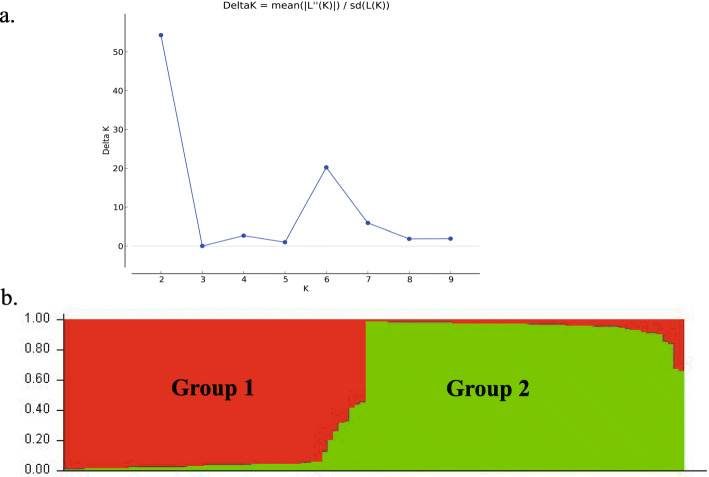
STRUCTURE analysis of the number of population for K. a. The number of subpopulations(k) was identified based on maximum likelihood and k values. The most likely value of k identified by STRUCTURE was observed at k = 2. Note: Red bands: Group 1, Green bands: Group 2. b. The proportion of each color reflects the probability that each of the test materials belongs the corresponding group

Based on the 39 alleles amplified using 14 InDels, the population structure of the 115 individuals was further estimated under the Hardy–Weinberg Equilibrium using the STRUCTURE V2.3.3 software. Delta K values were plotted against K values, and the best number of clusters was obtained via the Structure Harvester platform (http://taylor0.biology.ucla.edu/structureHarvester/). As shown in Fig. 6, Delta K reached a maximum value at K = 2, which indicated that the 115 cultivars could be partitioned into two populations (Fig. 6).

**Fig. 7:**
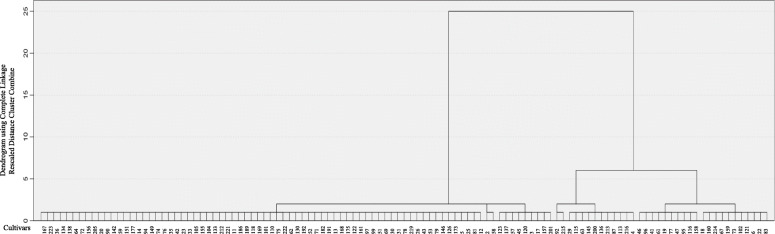
Dendrogram of cluster analysis for 115 Cannabis genotypes based on the flowering time

As showed in Table 1, the flowering time of 115 Cannabis genotype varied from 23 days to 125 days. Thereafter, cluster analysis was conducted using IBM SPSS Statistic 19.0 with the longest distance method and the Euclidean distance square. As shown in Fig. 7, at an inter-class distance of 25, the 115 genotypes were divided into two groups; group 1 included 34 cultivars, which mainly originated from the southern regions of (30), and group 2 contained 81 cultivars, most of which were from the northern regions of (74), such as Northwest China (15) and Northeast China (37).

**Table 1:** Information about the origins and flowering time for 115 Cannabis cultivars used in this study

Code	Name	Origin	Flowering Time(d)	Code	Name	Origin	Flowering Time(d)
2	620	NeiMengGu	23	97	723	NeiMengGu	35
3	627	YunNan	49	99	607	HeiLongJiang	35
4	Bendima1	ZheJiang	83	101	695	ShangDong	41
5	631	GanSu	31	102	Jingzhou3	LiaoNing	102
6	Yunma1	YunNan	119	104	108	HeiLongJiang	40
11	Fengma2	ShangXi	41	105	711	HeNan	44
12	634	GanSu	31	110	689	ShangXi	41
13	706	HeNan	35	113	661	HeiLongJiang	83
14	632	HeiLongJiang	37	115	625	YunNan	89
17	Lvliangma	ShangXi	56	116	710	YunNan	105
18	709	YunNan	108	118	659	HeiLongJiang	41
19	727	YunNan	97	120	676	ShanXi	48
20	114	NingXia	38	121	601	GuangXi	117
22	131	LiaoNing	101	122	644	NeiMengGu	35
23	106	ShangXi	36	123	Wuchang40	HeiLongJiang	51
25	636	HeiLongJiang	31	126	715	HeiLongJiang	31
26	690	HeNan	33	130	673	HeiLongJiang	34
29	698	YunNan	89	133	603	LiaoNing	40
30	694	HeiLongJiang	35	134	611	HeNan	39
31	720	AnHui	35	136	Yanji1	JiLin	79
33	707	JiLin	36	137	668	JiLin	51
35	717	AnHui	37	138	Yousha100	HeiLongJiang	39
36	724	ShangDong	39	142	606	NingXia	38
41	Nanhuadama	YunNan	97	145	608	YunNan	88
42	Fengma1	ShangXi	37	146	Yousha500	HeiLongJiang	32
43	617	JiLin	33	149	679	JiLin	37
45	626	GanSu	47	151	674	ShanXi	37
46	Dali8	YunNan	98	155	Neimengudali	NeiMengGu	45
47	637	ShangXi	101	156	716	HeiLongJiang	38
51	639	ZheJiang	35	157	Wandama3	Anhui	56
52	Hailun	HeiLongJiang	34	158	Jindan15	ChongQing	104
53	667	JiLin	33	159	660	YuNan	112
57	Jinma1	ShangXi	51	160	721	YunNan	108
58	105	ShanXi	25	161	117	GanSu	35
59	731	HeiLongJiang	38	167	677	HeiLongJiang	39
61	118	ZheJiang	97	168	732	HeNan	35
62	672	AnHui	43	169	101	HeiLongJiang	41
63	713	YunNan	88	173	666	HeiLongJiang	31
64	Qingdama1	HeiLongJiang	39	175	662	HeiLongJiang	35
67	708	YunNan	112	177	725	HeiLongJiang	37
69	610	HeNan	35	182	642	HeiLongJiang	35
71	115	AnHui	34	186	722	LiaoNing	41
72	697	GanSu	39	189	613	XinJiang	41
73	675	YunNan	114	191	712	HeiLongJiang	35
74	726	XinJiang	37	192	604	GanSu	34
75	705	HeNan	42	200	714	JiangSu	87
76	112	JiLin	37	201	Linlixiaoma	ChongQing	61
77	615	GuangXi	96	205	649	HeiLongJiang	38
78	641	HeiLongJiang	33	212	696	ShangDong	41
79	669	NeiMengGu	32	213	Yangquma	ShangXi	79
81	DaqingCK	HeiLongJiang	31	214	HL512	HeiLongJiang	99
83	Yunma6	YunNan	125	215	624	YunNan	71
87	104	YunNan	79	216	671	YunNan	83
90	702	HeiLongJiang	38	219	109	HeiLongJiang	33
92	628	AnHui	69	221	647	JiLin	41
94	622	HeiLongJiang	37	222	L1	ShangDong	42
95	116	JiangSu	101	223	L2	ShangDong	39
96	Bama2	GuangXi	98

### Screening of sex-linked InDel markers and PCR-based verification of known-sex plants

**Fig. 8:**
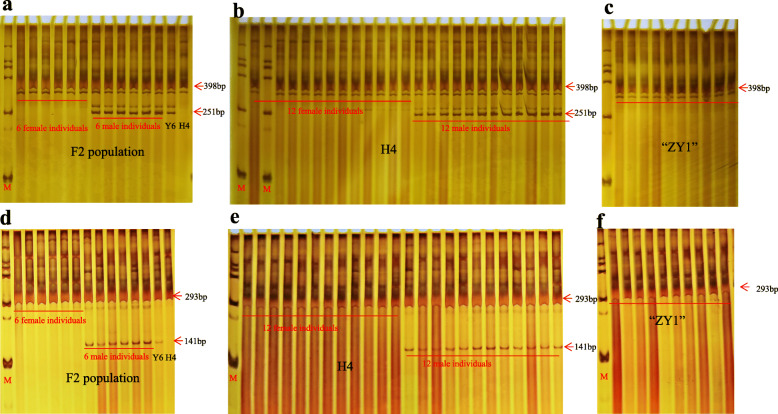
Amplification results of the two pairs of primers in F_2_ population crossed using “Y6” and “H4”, a dioecious variety “H4”and feminized variety,“ZY1”. a, b, c: Primer Cs-I1–10; d, e, f: Cs-I1–15. M: marker DL2000

Based on the latest report which indicated that chromosome pair 1 was the sex chromosome pair in Cannabis [34], fifteen pairs of primers evenly distributed on chromosome 1 were designed and used to amplify twelve samples (six females and six males) from the F_2_ population crossed by “Yunma 6” and “H4” (Table S2). As shown in Fig. 8a and Fig. 8d, two primers pairs (Cs-I1–10, Cs-I1–15) amplified two bands in male plants (251 bp and 398 bp; 293 bp and 141 bp), while one band (398 bp; 293 bp) was amplified in female plants.

To further verify the versatility and accuracy of the two primers pairs, samples from 24 known-sex plants from the dioecious variety, “H4”, and 10 known-sex plants from the feminized variety, “ZY1” were used for amplification via PCR, respectively. The results showed that 12 female plants showed amplification of the 398-bp fragment, while 12 male plants showed amplification of the two bands (398 bp and 251 bp in size) (Fig. 8b). Consistent with the amplification fragment in female plants of “H4”, all plants from “ZY1” showed amplification of the 398-bp fragment using the Cs-I1–10 primer pair (Fig. 8c). Similar to the Cs-I1–10 primer pair, 12 female plants and all plants from “ZY1” showed amplification of the 293-bp fragment, while 12 male plants showed amplification of the two bands (293 bp and 141 bp in size) using Cs-I1–15 primer pair (Fig. 8e-f).

## Discussion

Although different types of molecular markers, such as RAPD, ISSR, SSR, SNP and ARFP, have been used in the molecular biology studies conducted on Cannabis, such as genetic diversity analysis, sex identification, and QTL mapping [9, 10, 12, 15, 30, 31], these molecular makers remain fewer in number compared with those available for other crops, which poses challenges for genetic map construction and QTL mapping. In addition, a genome-wide survey of InDels has not yet been carried out for Cannabis. In this study, 31,802 InDel markers were identified in the Cannabis genome, and the average density across the FN genome was 0.031 InDels/kb (Table S1), which was much less compared to that found in other species such as rice, oilseed rape, maize and cotton [18, 22, 35, 36].

Molecular analyses like map-based gene cloning, GWAS, and MAS, rely on the availability of several genetic markers with detailed information of their position on the genome. The PCR-based InDel markers are extensively applied during initial mapping to identify unknown genes in rice, maize, wheat, and other crops [22, 37–40]. However, due to a lack of availability of chromosome-scale genome assembly, information about their physical position on the chromosome is not available [9–15, 30], which hinders the comprehensive molecular analysis of Cannabis. In this study, 26,982 InDel markers were developed with a density of 26.7 InDels/Mb. Notably, the exact physical positions of all identified InDels on the Cannabis genome were also determined, rendering it convenient to identify InDel markers in target genome regions, which, in turn, would help accelerate map-based cloning and marker-assisted trait selection research in Cannabis.

To analyse the population structure of the 115 Cannabis germplasms from the varieties cultivated in China, 84 InDels distributed along the Cannabis chromosomes with intervals of approximately 10 Mb were selected for the polymorphism analysis, and 38 InDels were found to exhibit polymorphism among three accessions. The polymorphism rate was 45.2%, similar to the extent in chickpea (46.6%) [41], lower than that found in jute (58%) [26], and higher than that in maize (18.68%) [22], which indicated that the polymorphism rate might relate to the plant species. Additionally, of the 36 InDels, 14 InDels amplifying only two fragments were selected for the genotyping of the 115 accessions. The PIC values ranged from 0.1209 to 0.6351, with an average of 0.4109, indicating that most of the InDels have a moderate range of genetic diversity, lower than that of SSR markers in Cannabis [10]. The possible reason was that most InDels used in this study are single-locus (Fig. S2), while, in general, SSRs are multi-locus.

The genetic structure of different genotypes can guide breeding programs for developing varieties with a broad genetic background. The genetic diversity of the Cannabis germplasm has been analysed using two types of markers: SSR and ISSR [9, 30, 31]. In the present study, 39 fragments were amplified using the 14 InDels, and when Delta K was at a maximum value of 2, the 115 accessions were partitioned into two subgroups. In group 1, the sharing proportion of the cultivars of group 2 ranged from 0.011 to 0.453, while in group 2, its sharing proportion of group 1 varied from 0.011 to 0.336 (Table S3). Most cultivars from the northern regions of China belonged to Group I, while most cultivars from the southern regions belonged to Group II (Fig. 6). Similar to the results of population structure analysis, the 115 accessions were clearly clustered into two major groups using UPGMA clustering (Fig. 4). As Cannabis is an annual and photoperiod-sensitive crop, and the day length may determine the floral transition and flowering times, we suggest that the climate, influenced by the latitude and day length, is an essential factor affecting the Cannabis germplasm diversity. In this study, the 115 accessions from China were distinctly classified into two groups (Figs. 4, 5, 6 and 7), and the two groups were consistent with the temperate climate and subtropical climate zones in China, respectively, which was in agreement with the analysis of Gao et al. (2014) and Zhang et al. (2018) [9, 42]. Additionally, both group I and group II included the cultivars from central regions of China like the HeNan provinces, implying that the breeders in these areas might frequently exchange Cannabis germplasm resources with the breeders from the northern or southern regions.

Cannabis is a short-day crop, which is sensitive to photoperiod. Flowering time is an important agronomic trait that affects cannabidiol (CBD) and fibre yield content. Consistent with the population structure analysis, PCoA analysis and UPGMA clustering results (Figs. 4, 5 and 6), 115 Cannabis genotypes were also clustered into two groups according to their flowering time. The cultivars of the group 1 mainly originated from Southern China. In contrast, group 2 was mainly composed of varieties from the northern regions of China (Fig. 7). In general, when the northern Cannabis cultivars are introduced to the southern regions, the plants will encounter early flowering. In this study, though the cultivars ‘22’ and ‘214’ originated from northern China, the plants did not encounter early flowering when cultivated in the southern regions of China (HuNan province), which might support the notion of a superior germplasm for developing wide adaptable Cannabis varieties according to day length.

Owing to the different economic values between female and male plants, a suitable ratio of females to males individuals is vital for enhancing economic efficiency. To overcome the difficulties of the accurate identification of sex through morphological methods before flowering, eight pairs of markers mainly consisted of RAPD markers were reported for sex identification in Cannabis [11–15]. However, these RAPD markers had a common shortcoming of poor repeatability and dominance. Additionally, the accuracy of 8 pair markers for sex identification was only validated by using natural populations, thus limiting its application in the Cannabis breeding program [11–15]. In this study, the two primer pairs, Cs-I1–10 and Cs-I1–15, were screened for sex identification, and except for the natural populations, an F1-segregated population and a feminized variety were used to verify its accuracy (Fig. 8). Thus, its applications are broader than those previously reported for sex identification in Cannabis breeding program. Interestingly, similar to the sex-linked SSR markers CS308 [14], the same fragments in size were presented in both female as well as male plants using Cs-I1–10 and Cs-I1–15, indicating these markers were not specific to the Y chromosome, which was different from the markers MADC1 to MADC3 on Y chromosome [11–13].

## Conclusion

In this study, we first developed 31,802 non-redundant InDel markers with a density of 31.5/Mb in the FN genome. Of these markers, 14 InDel markers could be used to divide the 115 Chinese Cannabis cultivars into two groups by genetic diversity analysis, population structure, and PCoA analysis. Additionally, two InDel markers, Cs-I1–10 and Cs-I1–15, related to female and male plant in Cannabis have been screened out. These genome-wide InDels and data on the genetic relationships of the Chinese Cannabis germplasm would serve useful in the further molecular analysis in Cannabis, and two sex-linked markers may provide accurate sex identification strategies at the early stage of Cannabis in production and breeding program.

## Materials and methods

### Plant materials and DNA extraction

A total of 115 Cannabis accessions were collected from different regions in China and preserved in our institute. Detailed information on these cultivars is summarised in Table 1. Flowering time is the time from sowing to flowering. When more than 50% of the plants of each cultivar bloom, the flowering time was scored and listed in Table 1. Additionally, six female and six male individuals, selected from an F_2_ population derived from a cross between a female “H4” plant and a male “Yunma 6” (Y6) plant, were used for the screening of sex-linked marker. Furthermore, 24 samples (12 females and 12 males individuals) from the “H4” variety and ten samples from the feminized Cannabis variety, “ZY1”, were used for further validation of the sex-linked marker.

### DNA extraction

The young leaves of each sample at the flowering stage were collected for DNA extraction. A Plant Genomic DNA Kit (Tiangen Biotech, Beijing, China) was used for DNA extraction. DNA quality and quantity were checked using an Eppendorf BioSpectrometer (Eppendorf, Hamburg, Germany), and the DNA was further diluted to a 10 ng/L working solution.

### Detection and design of the insertions/deletions (InDels)

The genomic DNA sequences of PK and FN were obtained from ftp://ftp.ncbi.nlm.nih.gov/genomes/all/GCA/003/417/725/GCA_003417725.2_ASM341772v2. The DNA sequences of PK represented the reference genome, which was compared with that of FN using MUMer (http://mummer.sourceforge.net/manual/) software to capture the InDel loci (≥ 4 bp). Then, based on the InDel loci data, the primers were designed using the Primer 3.0 software (http://pgrc.ipk-gatersleben.de/misa/primer3.html). One pair of primers with the highest scoring was selected in the design results for the experiments. Furthermore, all InDel markers were checked for specificity using the TBtool software by blasting with the reference genome to avoid nonspecific amplification [43]. Only unique InDels were retained and listed in Table S1.

### InDel genotyping

The 84 primer pairs evenly distributed in the FN genome were selected for polymorphism analysis. Polymerase chain reactions (PCRs) were performed using 10 μL aliquots of the reaction mixture, including 7 μL of the PCR mix solution (Qingke, Nanjing, China), 1 μL of the forward primer (10 nmol/L), 1 μL of the reverse primer (10 nmol/L), and 1 μL of the DNA template. PCR was conducted as follows: an initial step at 95 °C for 5 min, followed by 32 cycles of 30 s at 94 °C, 30 s at 55 °C, 40 s at 72 °C, and a final extension of 10 min at 72 °C. Primers used for genotyping were listed in Table 2 and Table S2.

**Table 2:** The primers used in this study

Marker	Position	Forward Primer	Reverse Primer	Product (bp)	MAF	AlleleNo	GeneDiversity	PIC
I1–2	5,107,359	CCCTTGCACACTTATTTGACTAGT	CCATTGCTGTTTATATTCGGGTGG	232	0.5739	4.0000	0.5462	0.4635
I1–4	35,070,118	CGATACAATCTAAGGGGAGTAGGC	CTGAGAGTTAGCACCACCATTTTG	225	0.6261	4.0000	0.5072	0.4277
I1–6	55,100,664	TCAGTTAATAATCGCACGCACATC	GATCCTGGTTCGTGAAATTGATGG	215	0.4522	4.0000	0.6865	0.6351
I2–10	45,232,264	CTAACTAACCATCTACTGCGACCA	CTCTGGATCCATTTTCGTTTGAGG	217	0.4913	4.0000	0.6149	0.5407
I4–10	45,033,231	GTTCTAAGAGTGGATTCAACGAAGA	TTACAATTTCACCCCTGCTTAGTG	198	0.5435	4.0000	0.6027	0.5376
I5–6	25,186,057	GACTTTGACACCATTCGAGTTCAG	GTGTTTACCCCTTCTCACATAGGT	129	0.9348	4.0000	0.1243	0.1209
I5–9	40,002,310	TCATACTACTCTCCTGACCTCTCC	AATTGTGATGTTTTCTTGGAGGGC	287	0.7870	4.0000	0.3609	0.3362
I6–2	5,003,421	GGATAAATCTCCGAAATGCACTCT	GACAAGGTGATTTTGAAGAGTGGG	196	0.9217	3.0000	0.1470	0.1412
I6–6	25,003,694	TGGGCGAACTCAAGGTCAATATTA	CCTCTAGGCCTTCTCAGCTTAATT	157	0.7739	4.0000	0.3774	0.3487
I6–7	30,129,906	GTCTACAACATCTCCTCCACTCTC	ATTAAAATAGCCGCACGAAGAG	296	0.7000	4.0000	0.4429	0.3770
I6–8	35,088,175	TTTTGCTACTGGGAATTAGGCGAA	CAGAGGAGTCCAAGGAAGAAGAAA	280	0.4478	3.0000	0.6379	0.5629
I7–4	15,048,834	AAAATCCCAACCACACCGACC	CCACCACATCAAACCATTCAGATT	272	0.5652	3.0000	0.5255	0.4259
I8–2	5,036,397	AGCTCAATCTGCCCTTAGTTCTAC	GTTCATGTTCTCTTCCTCTCCTGT	224	0.4957	3.0000	0.5605	0.4634
I8–4	15,041,707	TACTGCAGGATATGTGTAAAGCGT	CACAATATGGGAGGAACAACAAGT	286	0.7652	5.0000	0.3950	0.3721

MAF: Major Allele Frquency; PIC: Polymorphism Information Content

### Genetic diversity assay and population structure

Similar band types of 115 Cannabis cultivars on the electropherograms amplified using the same InDel markers were considered the same allele. Each polymorphic band detected by the same given primer represented an allelic mutation. To generate molecular data matrices, clear bands for each fragment were scored in every accession for each primer pair and recorded as 1 (presence of a fragment), 0 (absence of a fragment), and 9 (complete absence of band). PowerMarker version 3.25 was used to calculate the polymorphism information content (PIC), the number of alleles (NA), major allele frequency (MAF), and gene diversity for each InDel. A clustering map was conducted based on genetic distances and the unweighted pair group method with arithmetic mean (UPGMA) using the SM functionality of the NTSYS-pc2.10e software. Principal Coordinate Analysis (PCoA) was also performed using the NTSYS-pc2.10e software to resolve clustering patterns among genotypes. STRUCTURE v2.3.4 was used to estimate the population structure of the 115 Cannabis genotypes, and the number of the sub-population (K) was set from 1 to 10 based on admixture models and correlated with band frequencies three times. IBM SPSS Statistic 19.0 was used for cluster analysis of 115 Cannabis cultivars with the longest distance method and the Euclidean distance square based on the flowering time of each cultivar.

## Supplementary Information

**Additional file 1.0 Table S1.** All InDel markers developed in this study.

**Additional file 2. Table S2.** The primers used for screenning of sex-linked InDel markers. Table S3. The genetic admixture of 115 cultivars. Fig. S1 The physical location of 84 InDel primers on Cannabis chromosome used in this study. Fig. S2. Amplification products from 96 Cannabis cultivars using the InDel markers CS-I1–2.

## Data Availability

Male-specific sequences of Cannabis cultivar ‘H4’ amplified by InDel marker Cs-I1–10 and Cs-I1–15 have been uploaded to the NCBI SRA database. SRA accession: PRJNA734672. The data will be accessible with the following link: “https://www.ncbi.nlm.nih.gov/sra/PRJNA734672”.

## Abbreviations

InDel: Insertion/Deletion
CBD: Cannabinoid
THC: tetrahydrocannabinol
RFLP: Restriction fragment length polymorphism
RAPD: Random amplified polymorphic DNA
AFLP: Amplification fragment length polymorphism
SSR: Simple sequence repeats
ISSR: Inter-simple sequence repeat
PCR: Polymerase chain reactions
PIC: Polymorphism information content
NA: Number of allele
MAF: Major allele frequency
UPGMA: Unweighted pair group method with arithmetic mean
