# Correction to: Genome-wide development of insertiondeletion (InDel) markers for Cannabis and its uses in genetic structure analysis of Chinese germplasm and sex-linked marker identification

**DOI:** 10.1186/s12864-021-07960-0

**Published:** 2021-09-13

**Authors:** Gen Pan, Zheng Li, Siqi Huang, Jie Tao, Yaliang Shi, Anguo Chen, Jianjun Li, Huijuan Tang, Li Chang, Yong Deng, Defang Li, Lining Zhao

**Affiliations:** 1 Institute of Bast Fiber Crops, Chinese Academy of Agricultural Sciences, Changsha, 410205 China; 2 Key Laboratory of the Biology and Process of Bast Fiber Crops, Ministry of Agriculture, Changsha, China

**Correction to: BMC Genomics 22, 595 (2021)**

**https://doi.org/10.1186/s12864-021-07883-w**

Following publication of the original article [1], it was reported that Fig. 2 was a duplicate of Fig. 3. The correct Fig. 2 is provided in this Correction article and the original article has been updated.

**Fig. 2:**
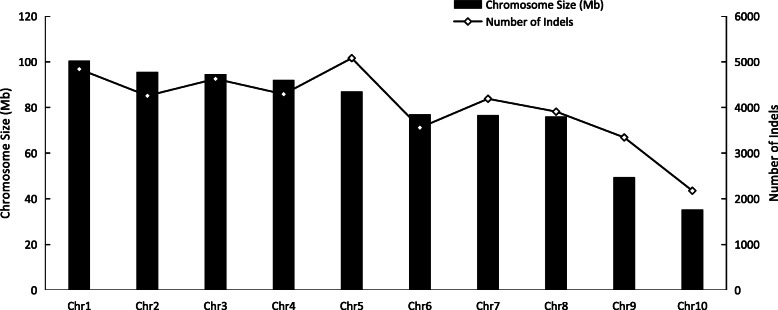
The number of Insert/Deletion (InDel) repeats on 10 chromosomes of Cannabis genome

## References

[1] Pan G, Li Z, Huang S (2021). Genome-wide development of insertion-deletion (InDel) markers for Cannabis and its uses in genetic structure analysis of Chinese germplasm and sex-linked marker identification. BMC Genomics.

