# Supplementary material for: Genome-wide development of insertion-deletion (InDel) markers for Cannabis and its uses in genetic structure analysis of Chinese germplasm and sex-linked marker identification
Source: BMC Genomics. 2021 Aug 5;22:595. doi: 10.1186/s12864-021-07883-w (PMC8340516; doi:10.1186/s12864-021-07883-w)
Supplement: Supplementary file 2 — Additional file 2. Table S2. The primers used for screenning of sex-linked InDel markers. Table S3. The genetic admixture of 115 cultivars. Fig. S1 The physical location of 84 InDel primers on Cannabis chromosome used in this study. Fig. S2. Amplification products from 96 Cannabis cultivars using the InDel markers CS-I1–2. [file 12864_2021_7883_MOESM2_ESM.doc]

Support Information

Table S1. The genome-wide InDel marker database developed in this study;

Table S2. The primers used for screenning of sex-linked InDel markers

Table S3. The genetic admixture of 115 cultivars

Fig. S1 The physical location of 84 InDel primers on Cannabis chromosome used in this study;

Fig. S2. Amplification products from 96 Cannabis cultivars using the InDel markers CS-I1-2

Table S2 The primers used for screening of sex-linked Indel markers

| Name | Chromosome | Position | FORWARD PRIMER1 (5'-3') | REVERSE PRIMER1 (5'-3') |
| --- | --- | --- | --- | --- |
| Cs-I1-1 | Chr1 | 5107359 | CCCTTGCACACTTATTTGACTAGT | CCATTGCTGTTTATATTCGGGTGG |
| Cs-I1-2 | Chr1 | 15085501 | GACAAGTTGAAGGTTTTAGGAGGC | TGAACAACTCTTCACACCTTGTAG |
| Cs-I1-3 | Chr1 | 25146673 | TCCTCGTTGAATCCACTCTTACAA | CGCTAAGTGTTTTAAGGGGCAAAT |
| Cs-I1-4 | Chr1 | 35070118 | CGATACAATCTAAGGGGAGTAGGC | CTGAGAGTTAGCACCACCATTTTG |
| Cs-I1-5 | Chr1 | 45016405 | CATTCCACTATGTTGACGATACTGT | AGCATAGGTGATAACTGTTGGGTT |
| Cs-I1-6 | Chr1 | 55100664 | TCAGTTAATAATCGCACGCACATC | GATCCTGGTTCGTGAAATTGATGG |
| Cs-I1-7 | Chr1 | 65257249 | TTGGGCTCATGTGTTGTTTGATAC | AGACAACCAACCCATACCAAGAAT |
| Cs-I1-8 | Chr1 | 75056780 | TCAACCAACACACTTCAATCCATG | GAGTTTTGTGTTTCTGTGGCTGTA |
| Cs-I1-9 | Chr1 | 85080432 | CAAAGGACTTAGACCCAGAACTGA | GGGTTTAGATCTCAGTCTGGGTTT |
| Cs-I1-10 | Chr1 | 90191387 | ACCTAACGATTTCCAAAAGC | ACCAAACGTTCTTTCTTTGC |
| Cs-I1-11 | Chr1 | 100584507 | TGAGGGAAGGGTATTTTGTCAA | TCTGTCTTAACACAAATCCGACCT |
| Cs-I1-12 | Chr1 | 23453127 | GCTCTTTCTCGTGTCCATTGAATC | ATGAGTTGCACTGTTGAAAGATCC |
| Cs-I1-13 | Chr1 | 16653927 | TGAAACGAACTACAACAAAAGGGG | AGTGTGAGGGAGCTTGTACATTTA |
| Cs-I1-14 | Chr1 | 8344395 | TAGGTATGGAGTATTGGTGGCATG | GAGATGAGTACCCCAAGAACAAGT |
| Cs-I1-15 | Chr1 | 17744322 | ATCTTTGAACTGCATATCC | AAACATACTCTCTATGC |

Table S3 The genetic admixture of 115 cultivars

| Cultivars | Group 1 (Proportion) | Group 2 (Proportion) | Cultivars | Group 1(Proportion) | Group 2 (Proportion) | Cultivars | Group 1 (Proportion) | Group 2 (Proportion) |
| --- | --- | --- | --- | --- | --- | --- | --- | --- |
| 6 | 0.99 | 0.01 | 45 | 0.954 | 0.046 | 130 | 0.036 | 0.964 |
| 18 | 0.99 | 0.01 | 120 | 0.954 | 0.046 | 35 | 0.035 | 0.965 |
| 159 | 0.989 | 0.011 | 145 | 0.953 | 0.047 | 136 | 0.035 | 0.965 |
| 67 | 0.987 | 0.013 | 110 | 0.952 | 0.048 | 90 | 0.034 | 0.966 |
| 46 | 0.985 | 0.015 | 222 | 0.952 | 0.048 | 192 | 0.034 | 0.966 |
| 41 | 0.984 | 0.016 | 69 | 0.949 | 0.051 | 52 | 0.033 | 0.967 |
| 47 | 0.984 | 0.016 | 142 | 0.947 | 0.053 | 173 | 0.033 | 0.967 |
| 63 | 0.984 | 0.016 | 216 | 0.937 | 0.063 | 221 | 0.032 | 0.968 |
| 61 | 0.983 | 0.017 | 223 | 0.937 | 0.063 | 43 | 0.028 | 0.972 |
| 160 | 0.982 | 0.018 | 87 | 0.874 | 0.126 | 14 | 0.026 | 0.974 |
| 95 | 0.981 | 0.019 | 101 | 0.799 | 0.201 | 36 | 0.025 | 0.975 |
| 115 | 0.979 | 0.021 | 77 | 0.737 | 0.263 | 59 | 0.025 | 0.975 |
| 212 | 0.978 | 0.022 | 161 | 0.68 | 0.32 | 23 | 0.024 | 0.976 |
| 73 | 0.977 | 0.023 | 62 | 0.676 | 0.324 | 157 | 0.024 | 0.976 |
| 92 | 0.977 | 0.023 | 3 | 0.582 | 0.418 | 189 | 0.024 | 0.976 |
| 97 | 0.977 | 0.023 | 42 | 0.562 | 0.438 | 213 | 0.024 | 0.976 |
| 158 | 0.977 | 0.023 | 17 | 0.547 | 0.453 | 75 | 0.023 | 0.977 |
| 4 | 0.976 | 0.024 | 5 | 0.336 | 0.664 | 78 | 0.023 | 0.977 |
| 51 | 0.976 | 0.024 | 214 | 0.322 | 0.678 | 99 | 0.023 | 0.977 |
| 116 | 0.975 | 0.025 | 126 | 0.158 | 0.842 | 133 | 0.022 | 0.978 |
| 83 | 0.972 | 0.028 | 81 | 0.146 | 0.854 | 138 | 0.022 | 0.978 |
| 96 | 0.972 | 0.028 | 169 | 0.097 | 0.903 | 177 | 0.022 | 0.978 |
| 200 | 0.972 | 0.028 | 137 | 0.091 | 0.909 | 104 | 0.021 | 0.979 |
| 20 | 0.97 | 0.03 | 113 | 0.087 | 0.913 | 13 | 0.02 | 0.98 |
| 201 | 0.97 | 0.03 | 219 | 0.08 | 0.92 | 22 | 0.02 | 0.98 |
| 151 | 0.968 | 0.032 | 53 | 0.068 | 0.932 | 31 | 0.02 | 0.98 |
| 121 | 0.963 | 0.037 | 118 | 0.065 | 0.935 | 64 | 0.02 | 0.98 |
| 215 | 0.962 | 0.038 | 167 | 0.062 | 0.938 | 72 | 0.019 | 0.981 |
| 12 | 0.961 | 0.039 | 102 | 0.052 | 0.948 | 149 | 0.019 | 0.981 |
| 79 | 0.961 | 0.039 | 30 | 0.049 | 0.951 | 205 | 0.019 | 0.981 |
| 19 | 0.96 | 0.04 | 146 | 0.048 | 0.952 | 122 | 0.018 | 0.982 |
| 134 | 0.96 | 0.04 | 94 | 0.046 | 0.954 | 156 | 0.018 | 0.982 |
| 2 | 0.958 | 0.042 | 33 | 0.045 | 0.955 | 123 | 0.015 | 0.985 |
| 26 | 0.958 | 0.042 | 76 | 0.043 | 0.957 | 175 | 0.015 | 0.985 |
| 71 | 0.958 | 0.042 | 74 | 0.04 | 0.96 | 168 | 0.014 | 0.986 |
| 57 | 0.957 | 0.043 | 11 | 0.039 | 0.961 | 155 | 0.013 | 0.987 |
| 29 | 0.956 | 0.044 | 25 | 0.039 | 0.961 | 191 | 0.012 | 0.988 |
| 58 | 0.955 | 0.045 | 186 | 0.038 | 0.962 | 182 | 0.011 | 0.989 |
| 105 | 0.955 | 0.045 |  |  |  |  |  |  |


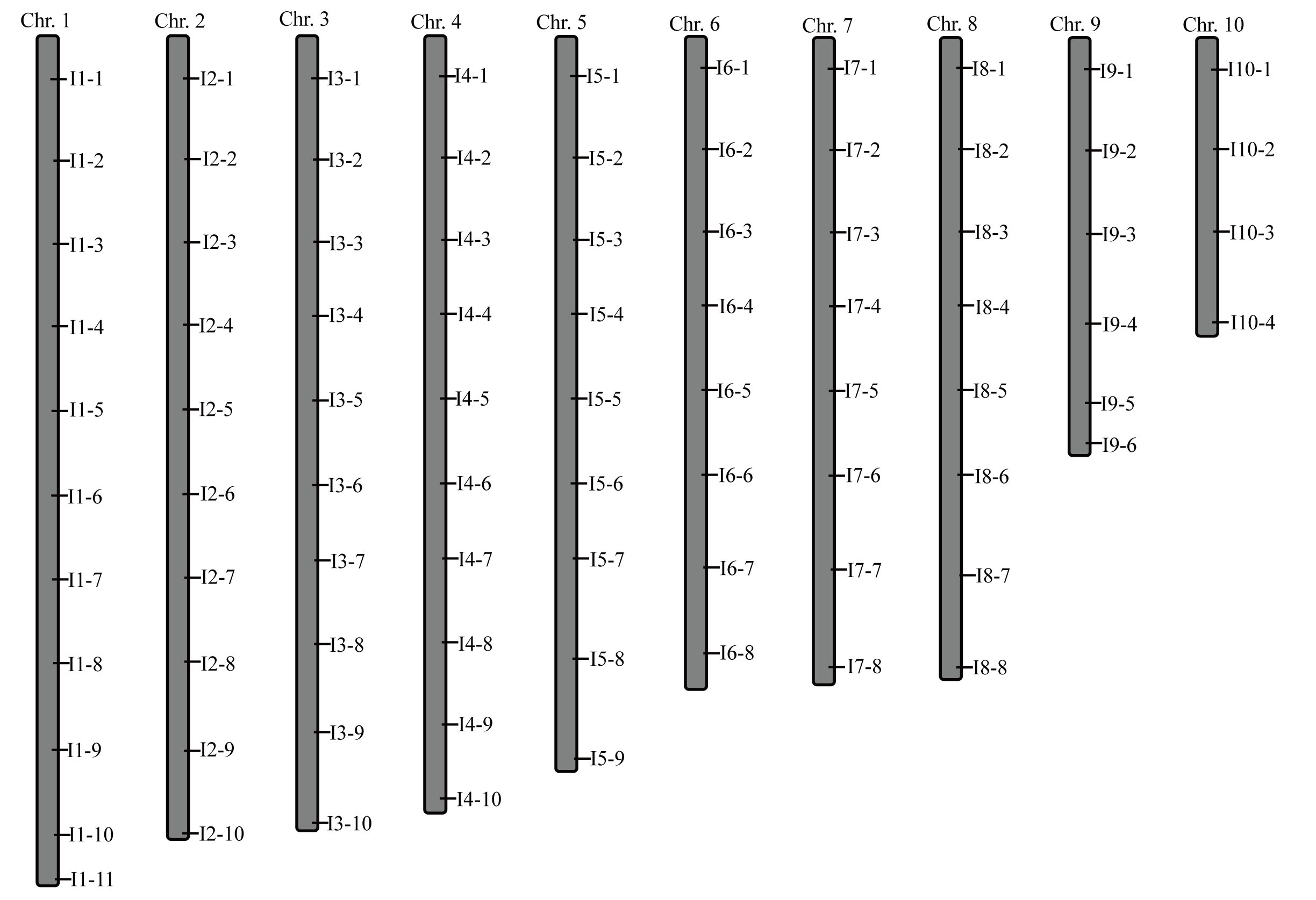


**Fig. S1** The physical location of 84 InDel primers on Cannabis chromosome used in this study


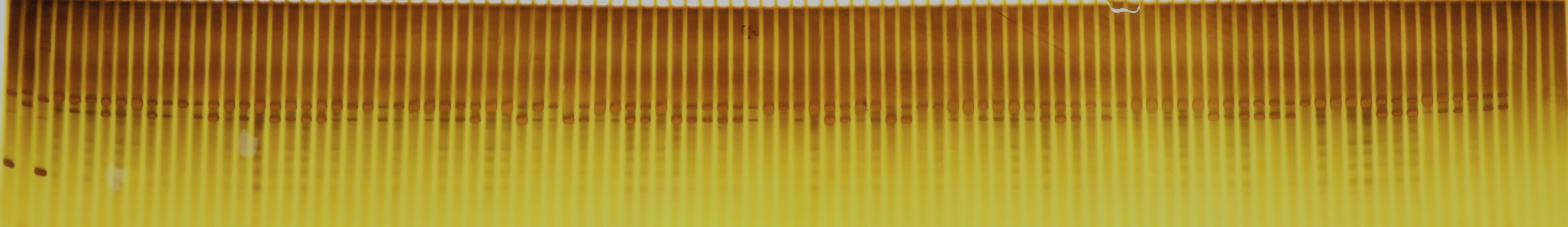


M

M

**Fig. S2** Amplification products from 96 Cannabis cultivars using the Indel markers I1-2

M：500 bp marker
